# Assessment of Treg-related lncRNAs in epilepsy

**DOI:** 10.3389/fnmol.2022.1031314

**Published:** 2023-01-26

**Authors:** Guive Sharifi, Reyhane Eghtedarian, Mohammad Taheri, Bashdar Mahmud Hussen, Solat Eslami, Soudeh Ghafouri-Fard, Arezou Sayad

**Affiliations:** ^1^Skull Base Research Center, Loghman Hakim Hospital, Shahid Beheshti University of Medical Sciences, Tehran, Iran; ^2^Phytochemistry Research Center, Shahid Beheshti University of Sciences, Tehran, Iran; ^3^Urology and Nephrology Research Center, Shahid Beheshti University of Medical Sciences, Tehran, Iran; ^4^Institute of Human Genetics, Jena University Hospital, Jena, Germany; ^5^Department of Pharmacognosy, College of Pharmacy, Hawler Medical University, Erbil, Iraq; ^6^Center of Research and Strategic Studies, Lebanese French University, Erbil, Iraq; ^7^Department of Medical Biotechnology, School of Medicine, Alborz University of Medical Sciences, Karaj, Iran; ^8^Dietary Supplements and Probiotic Research Center, Alborz University of Medical Sciences, Karaj, Iran; ^9^Men's Health and Reproductive Health Research Center, Shahid Beheshti University of Medical Sciences, Tehran, Iran; ^10^Department of Medical Genetics, School of Medicine, Shahid Beheshti University of Medical Sciences, Tehran, Iran

**Keywords:** epilepsy, lncRNA, TH2-LCR, RMRP, IFNG-AS1 (NEST), MAFTRR, FLICR

## Abstract

Recent studies have shown dysregulation of several groups of long non-coding RNAs in the context of epilepsy. According to evidence regarding the role of regulatory T cells in this disorder, we examined expression levels of regulatory T cell-related lncRNAs, namely TH2-LCR, RMRP, IFNG-AS1 (NEST), MAFTRR and FLICR in the blood of epileptic cases compared with controls. Expression of RMRP was lower in patients with refractory epilepsy compared with controls [expression ratio (95% CI) = 0.32 (0.13–0.8), adjusted *p*-value = 0.0008]. Besides, its expression was lower in refractory patients vs. non-refractory patients [expression ratio (95% CI) = 0.2 (0.1–0.41), adjusted *p*-value < 0.0001]. Expression of TH2-LCR was lower in refractory patients vs. controls [expression ratio (95% CI) = 0.4 (0.17–0.93), adjusted *p*-value = 0.0044] and in refractory patients vs. non-refractory ones [Expression ratio = 0.28 (0.19–0.58), *p*-value < 0.0001]. Expression of NEST was higher in total patients [expression ratio (95% CI) = 2.48 (1.15–5.27), adjusted *p*-value = 0.0012] and in both groups of patients compared with controls. However, its expression was not different between refractory and non-refractory cases. Similarly, FLICR and MAFTRR were over-expressed in total cases and both groups of patients compared with controls, but their expressions were similar between refractory and non-refractory cases. MAFTRR could differentiate between total epileptic cases and controls with AUC value of 0.8. This lncRNA could separate refractory and non-refractory cases from healthy controls with AUC values of 0.73 and 0.88, respectively. This study provides evidence for deregulation of regulatory T cell-related lncRNAs in epilepsy and their potential role as diagnostic markers in this condition.

## Introduction

Epilepsy is a complex condition with several known and unknown background, among them being abnormal activity of the immune system. Immune activation that results in functional rewiring of the brain has been shown to be the cause or effect of seizure (Xu et al., 2021). A bulk of evidence has revealed the functional roles of regulatory immune cells in the modulation seizures as well as in the epileptogenic processes (Xu et al., 2021). CD4 + FoxP3+ regulatory T cells (Tregs) have a particular role in this condition. Depletion of brain Tregs can lead to exacerbation of hippocampal neuroinflammation and induction of oxidative stress following status epilepticus (Yue et al., 2022). Moreover, it can reduce survival hippocampal neurons and aggravate seizure activity. Therefore, amplification of brain Tregs has been regarded as an antiepileptic strategy in animal models (Yue et al., 2022). Another study in rat models of temporal lobe epilepsy has shown the important roles of Th17, Tregs, and related cytokines in this disorder and suggested the efficacy of balancing Th17 and Tregs as a therapeutic option for patients with epilepsy (Wei et al., 2022).

In addition, a group of studies has highlighted contribution of long non-coding RNAs (lncRNAs) in the epileptogenic processes (Hashemian et al., 2019; Villa et al., 2019). These epigenetic factors regulate the pathophysiological process of epilepsy and have been found to be dysregulated during epileptogenesis. Experiments in human subjects affected with epilepsy and in animal models of this disorder have shown abnormal expression of non-coding RNAs (Manna et al., 2022). Based on the importance of lncRNAs in the regulation of Treg functions (Luo and Wang, 2020), we have selected five Treg-related lncRNAs to assess their expression in patients with epilepsy. These lncRNAs are FLICR (FOXP3 Regulating Long Intergenic Non-Coding RNA), NEST (IFNG-AS1), RMRP (RNA Component of Mitochondrial RNA Processing Endoribonuclease), MAFTRR (MAF Transcriptional Regulator RNA) and TH2-LCR (Th2 Cytokine Locus Control Region).

## Materials and methods

### Enlisted subjects

Eighty epileptic patients and 50 normal individuals were recruited in the current study. Cases were diagnosed based on electroencephalogram and brain MRI. Those included in the non-refractory group had no seizures throughout 6 months prior to sampling. Patients with refractory seizure had seizures in spite of taking appropriate doses of at least three antiepileptic medications. The study was approved by the ethical committee of Shahid Beheshti University of Medical Sciences (IR.SBMU.RETECH.REC.1400.337). Informed consent was signed by all participants. Controls had no neurological, psychiatric or systemic diseases.

### Experiments

Total RNA was retrieved from whole blood samples using the RNJia Kit (ROJE Technologies, Tehran, Iran). Then, 50 ng of extracted RNA was used for production of cDNA using AddScript cDNA synthesis kit (AddBio, Korea). Expressions of FLICR, MAFTRR, NEST, RMRP and TH2-LCR were quantified in patients and controls using Ampliqon master mix (Denmark). Reactions were performed in StepOnePlus Real-Time PCR System. B2M was used as normalizer (reference gene for qPCR) in a way that transcript levels of all genes were calculated in relation with expression levels of B2M in each sample. Information about primers is shown in Table 1.

**Table 1:** Primer sequences and corresponding amplified regions.

Gene	Sequence 5 → 3	Primer length (bp)
*B2M*	F-AGATGAGTATGCCTGCCGTG	20
	R-GCGGCATCTTCAAACCTCCA	20
*FLICR*	F-GGG CTT TTC CAG AAG GGT CT	20
	R-AGC CCA GGG TTC TAG TCG	18
*MAFTRR*	F-CTG AAG GGA CAG GAC GGA CAA C	22
	R-GGG GAA AAC CTG GAA AGA GGG A	22
*NEST*	F-AGC TGA TGA TGG TGG CAA TCT	21
	R-TGA CTT CTC CTC CAG CGT TTT	21
*RMRP*	F-GTA GAC ATT CCC CGC TTC CCA	21
	R-GAG AAT GAG CCC CGT GTG GTT	21
*TH2-LCR*	F-GCT CCC CAG GCT TTT GAG ATA	21
	R-TGG TGA TGC TGA AGG GAG AC	20

### Statistical analyses

GraphPad Prism version 9.0 (La Jolla, CA, United States) was used for statistical analysis. We compared expressions of five Treg-related LncRNA genes including TH2-LCR, RMRP, IFNG-AS1 (NEST), MAFTRR and FLICR in peripheral blood samples obtained from patients with epilepsy (40 refractory cases and 40 non-refractory cases) and 50 healthy controls. Expression levels in were calculated using the Efficiency adjusted Ct values. Expression levels in each sample were calculated using the Efficiency adjusted Ct of the normalizer gene (B2M)—Efficiency adjusted Ct of the target gene (comparative −ΔCt method). Normal/gaussian distribution of the values was assessed using the Shapiro–Wilk test. Differentially expressed genes were identified using Kruskal–Wallis test. This analysis was repeated for all comparisons (refractory epilepsy, non-refractory epilepsy, and total epilepsy patients vs. healthy controls).

Correlation between gene expression levels in patients and control samples was assessed using Spearman’s rank correlation coefficient since data was not normally distributed.

Receiver operating characteristic (ROC) curve was illustrated to appraise diagnostic power of expression levels of differentially expressed genes. *p*-value < 0.05 was considered as significant. This type of analysis was used for assessment of the performance of expression levels of genes as diagnostic tests. It functions as a simple graphical tool for showing the accuracy of a medical diagnostic test.

## Results

### General information

Table 2 shows the sex ratio of patients with refractory and non-refractory seizure.

**Table 2:** Sex ratio of cases with epilepsy.

Groups	Refractory group	Non-refractory group
Total, number (%)	40 (33.33)	40 (33.33)
Male, number (%)	17(29.31)	24 (41.38)
Female, number (%)	23 (37.1)	16 (25.8)

Age (mean ± SD) values of controls, refractory patients, and non-refractory patients were 33.93 ± 9.04, 36.75 ± 7.75, and 32.70 ± 11.89, respectively.

### Expression assays

**Figure 1:**
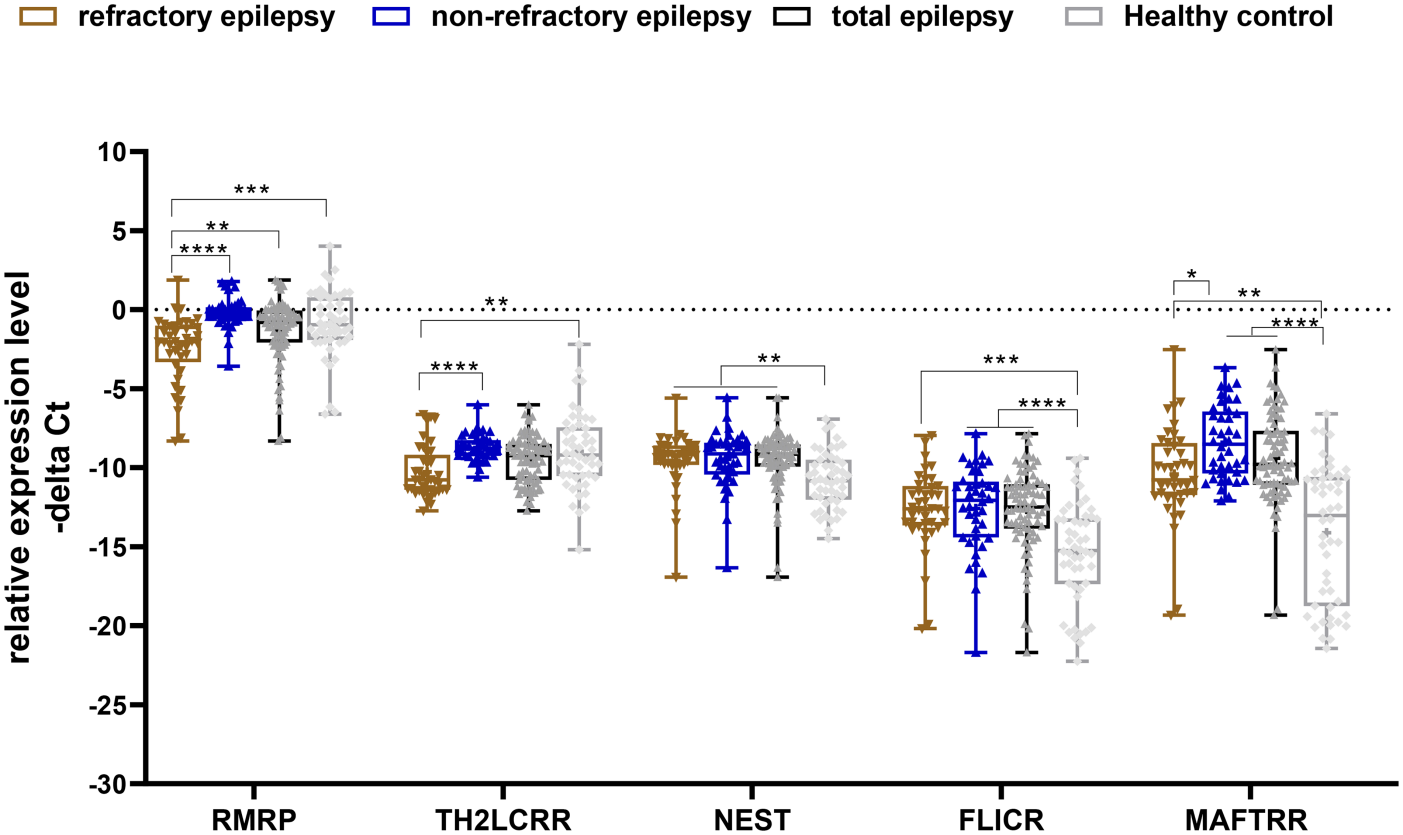
Relative expression levels of five Treg-related lncRNAs in epilepsy patients (total), refractory epilepsy, non-refractory epilepsy and healthy controls as described by −ΔCt values. −ΔCt Data were plotted as box and whisker plots showing the median [line], mean [cross], interquartile range [box], and minimum and maximum values. A non-parametric Kruskal–Wallis test was used to identify differentially expressed genes between patients (refractory epilepsy, non-refractory epilepsy and total epilepsy patients) and healthy controls (^*^p-values < 0.05, ^**^p-values < 0.01 ^***^p-values < 0.001, and ^****^p-values < 0.0001).

Significant differences were detected in expression of RMRP, TH-2LCR, NEST, MAFTRR, and FLICR between certain study subgroups (Figure 1).

Expression of RMRP was lower in patients with refractory epilepsy compared with controls [expression ratio (95% CI) = 0.32 (0.13–0.8), adjusted *p*-value = 0.0008]. Besides, its expression was lower in refractory patients vs. non-refractory patients [expression ratio (95% CI) = 0.2 (0.1–0.41), adjusted *p*-value < 0.0001]. Expression of TH2-LCR was lower in refractory patients vs. controls [expression ratio (95% CI) = 0.4 (0.17–0.93), adjusted *p*-value = 0.0044] and in refractory patients vs. non-refractory ones [Expression ratio = 0.28 (0.19–0.58), *p*-value < 0.0001]. Expression of NEST was higher in total patients [expression ratio (95% CI) = 2.48 (1.15–5.27), adjusted *p*-value = 0.0012] and in both groups of patients compared with controls. However, its expression was not different between refractory and non-refractory patients. Similarly, FLICR and MAFTRR were over-expressed in total cases and both groups of patients compared with controls, but their expressions were similar between refractory and non-refractory patients (Table 3).

**Table 3:** Results of expression study of five Treg-related lncRNAs, namely RMRP, TH-2LCR, NEST, MAFTRR, and FLICR in peripheral blood of patients with epilepsy (refractory and non-refractory groups) compared with healthy controls.

Studied genes		Total epilepsy patients vs. controls (80 vs. 50)	Refractory patients vs. controls (40 vs. 50)	Non-refractory patients vs. controls (40 vs. 50)	Refractory patients vs. non-refractory patients (40 vs. 40)
RMRP	Expression ratio (95% CI)	0.72 (0.34–1.48)	0.32 (0.13–0.8)	1.57 (0.83–1.56)	0.2 (0.1–0.41)
	Adjusted *P*-value	>0.9999	**0.0008**	0.1516	**<0.0001**
TH2-LCR	Expression ratio(95% CI)	0.69 (0.33–1.4)	0.4 (0.17–0.93)	1.18 (0.6–2.29)	0.28 (0.19–0.58)
	Adjusted *P*-value	>0.9999	**0.0044**	0.9499	**<0.0001**
NEST	Expression ratio (95% CI)	2.48 (1.15–5.27)	2.34 (1.1–4.94)	2.48 (1.15–5.27)	0.94 (0.42–2.12)
	Adjusted *P*-value	**0.0012**	**0.0021**	**0.0012**	>0.9999
FLICR	Expression ratio (95% CI)	6.77 (2.41–19.02)	6.58 (2.01–21.55)	6.96 (2.02–24.08)	0.94 (0.29–3.01)
	Adjusted *P*-value	**<0.0001**	**0.0003**	**<0.0001**	>0.9999
MAFTRR	Expression ratio (95% CI)	26.5 (7.21–96.8)	13.08 (2.82–60.67)	53.48 (13.62–208.51)	0.24 (0.07–0.8)
	Adjusted *P*-value	**<0.0001**	**0.0051**	**<0.0001**	0.0126

The expression ratio of each gene is shown as mean and 95% Confidence interval (95% CI). Numbers in column identifiers indicate the number of persons in each group being compared.The bold values mean a *p*-value is less than a 0.05 significance level.

Expression of NEST was correlated with expressions of TH2-LTR and RMRR in healthy controls (correlation coefficients = 0.32 and 0.35, respectively). Moreover, expression of FLICR was correlated with TH2-LCR expression in non-refractory and controls groups (correlation coefficients = 0.46 and 0.44, respectively) and with MAFTRR levels in non-refractory group (correlation coefficient = 0.31). Finally, expression of TH2-LCR was correlated with MAFTRR level in refractory and non-refractory groups (correlation coefficients = 0.71 and 0.37, respectively) and with RMRP level in non-refractory patients (correlation coefficient = 0.44; Table 4).

**Table 4:** Spearman’s correlations between expression levels of five lncRNA among refractory epilepsy patients (*N* = 40), non-refractory epilepsy patients (*N* = 40), and healthy controls (*N* = 50).

	FLICR			TH2LCR			MAFTRR			RMRP		
	Refractory	Non-refractory	Healthy controls	Refractory	Non-refractory	Healthy controls	Refractory	Non-refractory	Healthy controls	Refractory	Non-refractory	Healthy controls
NEST	0.08	0.22	0.21	−0.017	0.17	0.32^*^	−0.01	−0.01	0.1	0.55^**^	0.4	0.35^*^
FLICR				0.20	0.46^*^	0.44^*^	0.20	0.31^*^	0.07	0.069	0.14	0.07
TH2LCR							0.71^**^	0.37^*^	0.14	−0.24	0.44^*^	0.16
MAFTRR										−0.22	0.1	0.17

* *p* < 0.05,

** *p* < 0.001.

**Figure 2:**
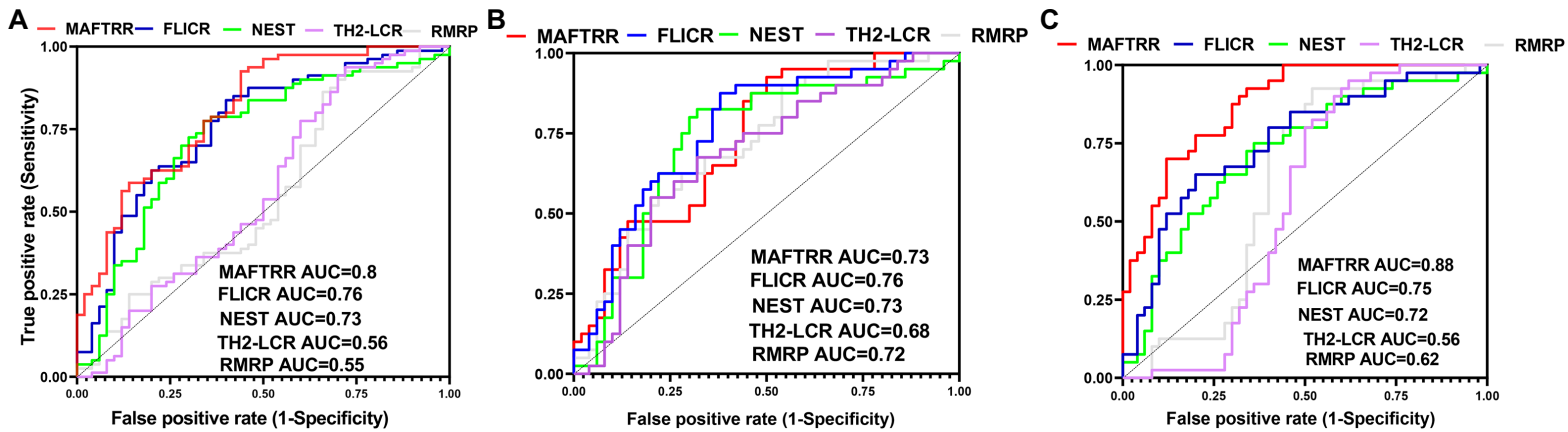
ROC curves of five Treg-related lncRNAs, namely RMRP, TH-2LCR, NEST, MAFTRR and FLICR in total epilepsy cases **(A)**, Refractory epilepsy patients **(B)**, and non-refractory epilepsy patients **(C)**.

MAFTRR could differentiate between total epileptic cases and controls with AUC value of 0.8. This lncRNA could separate refractory and non-refractory cases from healthy controls with AUC values of 0.73 and 0.88, respectively (Figure 2; Table 5).

**Table 5:** ROC curve analysis in patients with total epilepsy cases, refractory epilepsy patients, and non-refractory epilepsy patients.

	MAFTRR				FLICR				NEST				TH2-LCR				RMRP			
	AUC ± SD	Sensitivity	Specificity	*p*-value	AUC ± SD	Sensitivity	Specificity	*p*-value	AUC ± SD	Sensitivity	Specificity	*p*-value	AUC ± SD	Sensitivity	Specificity	*p*-value	AUC ± SD	Sensitivity	Specificity	*p*-value
Total cases vs. healthy normal controls (80 vs. 50)	0.8 ± 0.04	0.92	0.56	<0.000	0.76 ± 0.04	0.83	0.74	<0.00	0.73 ± 0.04	0.77	0.66	<0.000	0.56 ± 0.05	0.93	0.28	0.25	0.54 ± 0.05	0.92	0.28	0.35
Refractory patients vs. controls (40 vs. 50)	0.73 ± 0.05	0.92	0.5	0.0002	0.76 ± 0.05	0.87	0.62	<0.0001	0.73 ± 0.05	0.82	0.68	0.0002	0.68 ± 0.05	0.67	0.68	0.002	0.72 ± 0.05	0.9	0.46	0.0004
Non-refractory patients vs. healthy controls (40 vs. 50)	0.88 ± 0.03	0.92	0.66	<0.0001	0.75 ± 0.05	0.65	0.8	<0.0001	0.72 ± 0.05	0.75	0.64	0.0003	0.56 ± 0.06	0.95	0.38	0.29	0.62 ± 0.06	0.92	0.48	0.04

## Discussion

LncRNAs can affect pathophysiology of epilepsy through different mechanisms among them is regulation of immune responses (Zhao et al., 2022). Tregs have fundamental roles in the regulation of immune activity and are involved in the suppression of epileptogenesis (Xu et al., 2021). Therefore, Treg-related lncRNAs are putative candidates for identification of the underlying mechanism of epilepsy. In the current research project, we compared expressions of five of these lncRNAs between epileptic cases and healthy subjects.

Expressions of RMRP and TH2-LCR were lower in refractory patients vs. controls and vs. non-refractory ones. RMRP has been shown to be over-expressed in some immune-related conditions (Hussen et al., 2021). Expression of this lncRNA has been up-regulated in T cells of rheumatoid arthritis cases and it has been correlated with duration of the disorder (Moharamoghli et al., 2019). On the other hand, RMRP has been shown to be under-expressed in major depressive disorder cases in correlation with severity of disorder (Seki et al., 2019). RMRP is also involved in the regulation of neuron apoptosis since it can accelerate autophagy-mediated apoptosis of neurons *via* modulation of miR-3,142/TRIB3 axis (Tang et al., 2022). TRIB3 has a putative role in epileptogenesis, since its inhibition can protect against kainic acid-induced neurotoxic injuries (Zhang et al., 2019). Thus, RMRP contributes to the pathogenesis of epilepsy through immune-related and -unrelated mechanisms.

TH2-LCR is involved in the modulation of production of Th2 cytokines (Koh et al., 2010). The impact of cytokines in the epilepsy has been revealed for many years. Over-expression of IL-1β, TNF-α, and IL-6 have been reported in experimental models of seizures (Vezzani et al., 2008).

On the other hand, NEST, FLICR, and MAFTRR were over-expressed in total cases and both groups of patients compared with controls, but their expressions were similar between refractory and non-refractory patients. FLICR modulates expression of Foxp3 and facilitate development of a subgroup of Tregs with low expression of Foxp3 (Zemmour et al., 2017). NEST has a regulatory effect on methylation of the *IFN-G* locus; thus it modulates expression of IFN-γ (Gomez et al., 2013). In addition, NEST can reduce Th1-related proliferation of Tregs (Luo et al., 2017). IFN-γ has been among cytokines whose postictal and interictal levels have been higher in epilepsy patients compared with healthy subjects (Gao et al., 2017). Moreover, interictal IL-IFN-γ levels could predict severity of seizures (Gao et al., 2017).

Expression of NEST was correlated with expressions of TH2-LTR and RMRR in healthy controls. Moreover, expression of FLICR was correlated with TH2-LCR expression in non-refractory and controls groups and with MAFTRR levels in non-refractory group. Finally, expression of TH2-LCR was correlated with MAFTRR level in refractory and non-refractory groups and with RMRP level in non-refractory patients. This data shows a trend toward disease-specific pattern of correlation which can be different among refractory and non-refractory cases. Future studies are needed to show whether this pattern can affect response of patients to antiepileptic drugs.

MAFTRR could differentiate between total epileptic cases and controls with AUC value of 0.8. This lncRNA could separate refractory and non-refractory cases from healthy controls with AUC values of 0.73 and 0.88, respectively. Therefore, MAFTRR can be regarded as a diagnostic marker for epilepsy, particularly non-refractory epilepsy.

This study provides evidence for dysregulation of regulatory T cell-related lncRNAs in epilepsy and their potential role as diagnostic markers in this condition. Further studies are needed to assess expression of these ncRNAs in other biofluids and find possible correlations between their expression levels in the brain, lymphocyte, and saliva.

## Data availability statement

The original contributions presented in the study are included in the article/supplementary material, further inquiries can be directed to the corresponding authors.

## Ethics statement

The study protocol was approved by the ethical committee of Shahid Beheshti University of Medical Sciences (IR.SBMU.RETECH.REC.1400.337). The patients/participants provided their written informed consent to participate in this study.

## Author contributions

SG-F wrote the manuscript and revised it. MT and AS designed and supervised the study. BH, RE, and GS collected the data and performed the experiment. SE analyzed the data. All authors contributed to the article and approved the submitted version.

## Conflict of interest

The authors declare that the research was conducted in the absence of any commercial or financial relationships that could be construed as a potential conflict of interest.

## Publisher’s note

All claims expressed in this article are solely those of the authors and do not necessarily represent those of their affiliated organizations, or those of the publisher, the editors and the reviewers. Any product that may be evaluated in this article, or claim that may be made by its manufacturer, is not guaranteed or endorsed by the publisher.
